# Use of zinc sulfate for the development of zinc-fortified meat products from broiler meat

**DOI:** 10.5455/javar.2024.k864

**Published:** 2024-12-29

**Authors:** Asma Sultana, Nathu Ram Sarker, Raihan Habib, Md. Shahin Alam, Dulal Chandra Paul, Mst. Farhana Sharmin, Aminul Islam, Ruhul Amin, Md. Sazedul Karim Sarker

**Affiliations:** 1 Strengthening of Poultry Research and Development Project, Bangladesh Livestock Research Institute, Savar, Bangladesh; 2 Krishi Gobeshona Foundation, Dhaka, Bangladesh; 3 Department of Dairy Science, Bangladesh Agricultural University, Mymensingh, Bangladesh; 4 Animal Health Research Division, Bangladesh Livestock Research Institute, Savar, Bangladesh; 5 Poultry Production Research Division, Bangladesh Livestock Research Institute, Savar, Bangladesh

**Keywords:** Broiler, fortification, meatball, zinc

## Abstract

**Objective:**

The study aimed to assess the feasibility of incorporating food-grade zinc sulfate (ZnSO_4_) for making zinc-fortified meatballs from broiler meat.

**Materials and Methods:**

A total of 20 broiler breasts were treated with different concentrations of elemental zinc (Zn) of food-grade ZnSO₄ for making meatballs. The study included a control group without added Zn (T0: 0 mg/kg) and treatment groups with Zn concentrations of 10 mg/kg (T1), 20 mg/kg (T2), 30 mg/kg (T3), and 40 mg/kg (T4). Post-cooking, the meatball samples underwent preservation at −20°C for proximate composition, quality parameters, storage quality, texture, and sensory analysis.

**Results:**

Though the moisture content of meatballs did not differ significantly (*p* > 0.05), the T4 meatballs showed higher moisture percentages and significantly (*p* < 0.01) higher crude protein content compared to the control. However, a significant increase in cook yield (*p* < 0.01) and water-holding capacity (*p* < 0.05) were revealed by the T4 group. The T4 meatball recovered around 41.8% Zn. The malondialdehyde levels in all samples were acceptable when kept in the chiller for ten days. Moreover, the addition of varying doses of ZnSO_4_ did not result in a significant alteration (*p* > 0.05) in the hardness of the meatballs. The sensory panelists also validated the instrumental outcome of hardness.

**Conclusion:**

The addition of 40 mg/kg of elemental Zn from ZnSO_4_ improves nutritional composition and quality with no alteration of the texture and sensory properties of the meatball. This dose could be an effective strategy for fortifying chicken meat products and could offer a valuable source of dietary Zn for human consumption.

## Introduction

Zinc (Zn), the most essential mineral, supports a strong immune system in the human body. It is a crucial component in numerous enzymatic processes, significantly influencing the regulation of food intake, nutritional metabolism, protein synthesis, growth, and development [1]. The insufficiency of Zn is seen as a significant public health concern in the whole world. The children and adult population, especially women and senior people, are also experiencing Zn deficiency [2]. The Zn deficiency is also observed among preschool children in Bangladesh, standing at 44.6 percent, with the most significant prevalence observed among children residing in slum areas [2]. The daily Zn intake for preschool children residing in slums is reported to be 2.67 mg, whereas those in urban regions consume 3.23 mg [2]. It is worth noting that the recommended dietary allowance for Zn in this age group is 3–5 mg/day [3]. The factors lying behind this circumstance include food insecurity, a low socioeconomic status, inadequate consumption of animal-based protein sources, and high consumption of plant-based protein sources containing phytate, which hinders protein absorption in the human body.

Poultry is regarded as a crucial and promising sector in the livestock industry in Bangladesh, gathering increasing attention from domestic entrepreneurs and foreign investors. The demand for poultry products in Bangladesh has experienced substantial growth over the past decade. In Bangladesh, the broiler chicken population is estimated at around 525 million [4]. Among this population, roughly 60% (300–325 million) are raised on commercial farms, while the rest are reared in semi-scavenging or traditional rural backyard settings. Though the broiler sector has initiated efforts to enhance product variety and expand the value of its existing products by introducing other value-added offerings, still, there is still no product addressing public health dietary concerns. Only a limited number of companies are now addressing the issue of iron availability in their products. However, one of the most consumed meat-processed products in Bangladesh is meatballs, of which there are many varieties (including fishballs, beef, and chicken) available. Among these varieties, chicken meatballs are the most well-accepted and frequently eaten. The primary determinants of the ultimate product’s quality are the ingredients utilized in food preparation. These days, there is a sharp rise in demand for healthier chicken products. Such consumer requests have encouraged the development of meat product formulations containing a variety of healthy bioactive components.

Additionally, there needs to be more nutrient-sensitive, cost-effective, value-added products specifically targeting Zn because of its crucial role as a vital mineral for human health. According to Hess and Brown [5], foods fortified with zinc oxide (ZnO) and zinc sulfate (ZnSO₄) demonstrated good absorption. These two ingredients are also the least expensive fortifiers used in the food business. Although there was no difference in absorption from wheat fortified with either ZnSO_4 _or ZnO, water-soluble ZnSO_4_ is thought to be better absorbed than weakly soluble ZnO [6]. By European Regulation (EC) N. 1925/2006, ZnSO₄ and Zn gluconate are also permitted sources of Zn in meals and food supplements. ZnSO_4_ comprises around 23% elemental Zn, making it a viable contender for fortifying food products to address Zn deficiency [7]. There are some documents on Zn application to fortify dairy products and cereals. However, several works have been done on Zn biofortification of poultry meat. Probably, no report is available on using food-grade Zn to further process poultry products for improving the product nutritional quality.

Meat and meat products are primary sources of minerals with high bioavailability. Poultry meat is noted for high protein with a fairly stable composition of amino acids (alanine, asparagine, arginine, glutamine, leucine, lysine, etc.) and packed with minerals such as magnesium, potassium, phosphorus, and iron [8]. Fortifying chicken products with certain nutrients or elements, such as Zn, can offer significant nutritional advantages to a large population in Bangladesh. In order to effectively address the increasing domestic demand, it is imperative for the poultry sector in Bangladesh to substantially enhance its production. From this standpoint, researchers in the field of animal science are focusing their attention on developing specific poultry products that are enriched with nutrients and possess functional properties to promote human health. Simultaneously, enterprises attempt to expand their customer base by developing products and enhancing the value proposition of poultry products. In light of the current circumstances, the present study was undertaken to accomplish the objectives of 1) developing a Zn-fortified meat product by using broiler meat, 2) assessing the possibilities of using food-grade ZnSO_4_for producing Zn-fortified meat products without altering the quality and sensory attributes of the product, and 3) developing technology for Zn-fortified meat products for industrial use.

## Materials and Methods

### Ethical statement

All participants voluntarily participated and submitted informed consent forms before the study was conducted. Ethical approval for the involvement of human participants in this study was granted by the Institutional Review Board (IRB) of the Bangladesh Agricultural University Research System (BAURES), BAU, Mymensingh (approval number BAURES/ESRC/53/2024).

### Collection and processing of broiler meat

For conducting the study, a total of 20 broiler chickens were obtained from the local market. Carcasses were deboned, and collected breast meats were weighed, minced, and mixed. In order to produce Zn-fortified meatballs, the meat samples were subjected to treatment with five distinct levels of ZnSO₄ (zinc sulfate monohydrate) supplied by PD NAVKAR Bio-Chem Pvt. Ltd., India. These levels included 0 mg/kg (referred to as T0), 10 mg/kg (T1), 20 mg/kg (T2), 30 mg/kg (T3), and 40 mg/kg (T4). The dosages were chosen according to the elemental Zn for the human body. For 10, 20, 30, and 40 mg of elemental Zn, 27.5, 55, 82.5, and 110 mg were used in each kilogram as treatment. The flour and other ingredients were obtained from the local market.

### Preparation of Zn-fortified meatballs

The meatballs were prepared according to the study conducted by Ikhlas et al. [9], with some modifications. The ingredients used for meatball preparation were 65% chicken breast meat, 14.8% tapioca, 14% water, 1.8% salt, 2% sugar, 2.4% spices, and different levels of food-grade ZnSO_4_ for making different treatment groups.

The breast meat was diced into small portions and afterward subjected to freezing at a temperature of −20°C for a duration of 1 day. The frozen meat was minced and subsequently blended with flour, salt, spices, water, and varying amounts (0, 27.5, 55, 82.5, and 110 mg/kg) of ZnSO₄. The meatballs were manually produced, weighed, and subjected to a cooking temperature of 80°C for about 30 min until the core temperature reached 70°C. After completing the cooking process, the balls were immersed in distilled water ranging from 10°C to 15°C for a duration of 10 min. The meatballs were thereafter refrigerated at a temperature of −20°C for the purpose of analysis.

### Proximate composition

The moisture, protein, and fat content of the products were analyzed using the methods outlined by the Association of Official Analytical Chemists [10].

### pH

The pH values of the samples were determined using the methodology followed by Sultana et al. [11]. A meat sample weighing approximately 25 gm was homogenized with 100 ml of distilled water for a duration of 1 min using a mechanical blender. A 50-ml sample of the whole homogenate was promptly utilized for pH determination utilizing a digital pH meter (Jenway 3510 Standard Digital pH Meter Kit; 230 VAC/UK), following the calibration of the instrument with two standard buffers.

### Water-holding capacity (WHC)

The protocols of Lin and Huang [12] were modified for measuring the WHC of cooked meatball samples. It was determined by placing approximately 5 gm of the samples into a 50-ml centrifuge tube. Approximately 10 ml of distilled water was added, followed by a centrifugation (Thermo Scientific™ Sorvall™ ST 8 Small Benchtop Centrifuge) process at 2,000x *g* at 15°C for 10 min. The supernatant was carefully poured off, and the weight of the resulting sample was afterward measured. The calculation of WHC was performed in the following manner:

WHC = (final sample weight – original sample weight)/original sample weight. The higher WHC value (expressed by gm water absorbed/gm meat) indicates that more water was bound and, therefore, a higher water-holding ability of batter. The result was expressed as percentages.

### Cook yield

The cook yield was measured by measuring the change in sample weight before and after cooking, using the methodology outlined by Verma et al. [13]. The cooking yield was represented as a percentage. The cook yield can be calculated by multiplying the weight of the cooked meatballs by 100 and then dividing it by the weight of the uncooked meatballs.

### Texture analysis

The measurement of texture, namely, hardness, in meat products was conducted using a food texture analyzer (Brookfield’s CT3 food texture analyzer). To prepare the samples, frozen meatballs were kept at a temperature of 4°C–10°C for a duration of 24 h. The hardness of the samples was assessed after they were thawed. The experimental conditions included a trigger load of 0.067 N, a test speed of 5 mm/sec, a target value of 4.00 mm, a hold period of 0 sec, and a total of 2 cycles. After the 1st compression, the probe returned to the initial position and stopped for about 2 sec before the 2nd compression started. Only the hardness values were calculated.

### Thiobarbituric acid (TBA) analysis

The TBA levels of broiler meatballs were evaluated in triplicate using the 2-TBA method as outlined by Schmedes and Holmer [14], with minor adjustments. The values were determined on the 0th, 4th, 7th, and 10th days of preservation at a chilled temperature. The measurement of absorbance was conducted at a wavelength of 532 nm using a UV-VIS spectrophotometer (UV-1200, Shimadzu, Japan). The TBA levels were quantified in terms of milligrams of malondialdehyde (MDA) per kilogram of sample.

### Total viable count (TVC) analysis

The TVC of broiler meatball samples was determined on the 0th, 14th, and 28th days of preservation under freezing conditions at−20°C. The determination of TVC of bacteria was conducted using the methodology outlined by Wang et al. [15] with minor modifications. In this experiment, a meatball sample weighing 5 gm was mixed with 45 ml of sterile saline solution containing 0.9% sodium chloride. The mixture was then subjected to homogenization for a duration of 1 min. Subsequently, a serial ten-fold dilution using sterile saline solution was made, followed by plating 0.1 ml of the diluted sample onto plate count agar plates supplied by HIMEDIA(R) Pvt Ltd., India. The plates were subjected to incubation at a temperature of 36°C ± 1°C for a duration of 48 h in order to facilitate the enumeration of colony-forming units. The findings were quantified using the logarithmic scale of colony-forming units per gram (CFU/gm).

### Determination of amount of Zn

Zn amounts in meatball samples were determined from raw and cooked meatballs performed according to Kahraman and Ustunol [16], with some modifications. Five grams of sample was taken and treated with 25 ml of nitric acid to determine Zn in a meatball. After the reaction, 10 ml of perchloric acid was added and heated to concentrate. When the contained material turned yellowish or colorless, 2–3 ml of nitric acid was added and continued to be heated. After cooling, 2 ml of hydrochloric acid was added, and the fixed volume of measurement solution was prepared. The Zn content of samples was determined by atomic absorption spectrophotometer at the wavelength of 213.9 nm and calculated based on a standard curve.

### Sensory evaluation

The sensory evaluation was carried out in accordance with the methodology described by Sultana et al. [11]. The samples, both those treated with ZnSO₄ and those without, were subjected to sensory evaluation. There are eight distinct qualities taken into account, including tenderness, juiciness, flavor, sweetness, saltiness, astringent taste, mouthfeel, and overall acceptability. The evaluation of each character was conducted using a nine-point scale ranging from 1 to 9, with 1 representing a favorable trait and 9 representing an unpleasant trait. The panelists were requested to assess the meatball treated withZnSO_4_ in relation to its untreated counterpart.

### Statistical analysis

The experiment was replicated three times. The data obtained from the proximate composition, pH, cook yield, WHC, Zn amount, textural properties, sensory properties, and storage parameters (pH, color, TBA value, and microbiological properties) were analyzed by one-way analysis of variance using the Statistical Package for the Social Sciences (SPSS) statistical package program (SPSS Inc., USA). Significant differences among the means were compared using Duncan’s multiple range test (DMRT).

## Results and Discussion

### Proximate composition

Table 1 presents the proximate composition and quality parameters of meatballs produced from broiler meat exposed to different levels of ZnSO_4_ treatment. The results in Table 1 indicate no statistically significant differences (*p* > 0.05) in the moisture and ether extract percentages among the treatment groups when different levels of ZnSO_4_ were included in the meatballs. The meatballs’ moisture content and ether extract percentages exhibited a range of 72.00%–74.53% and 1.20%–2.10%, respectively. The results regarding moisture content are consistent with those from Huda et al. [17] study on commercial chicken meatballs. Huda et al. [17] observed that commercial chicken meatballs’ moisture and fat contents ranged from around 64.33% to 71.81% and from 4.26% to 14.00%, respectively. The findings of Ikhlas et al. [9] indicate that the moisture content in quail meatballs was 66.33%. This result was obtained through the use of 3% cassava flour in the manufacture of the meatballs. Nonetheless, a substantially more significant amount (18.18%) of crude protein was seen in the broiler meatballs that were treated with 40 mg/kg of elemental ZnSO_4_ (*p* < 0.01). However, according to Kahraman and Ustonol’s [16] findings, the Zn-fortified cheeses exhibited significantly greater protein content (*p* < 0.05) of 25.39% in comparison to the control group, which had a protein level of 24.62%. However, the increase in protein content in Zn-fortified meatballs might be due to the ability of Zn to interact with amino acid side chains, such as cysteine and histidine. This interaction resulted in improved protein folding and stability and aided in the retention of higher protein content.

### Quality parameters

The pH, cook yield, WHC, and hardness values for broiler meatballs are presented in Table 1. The pH value is a crucial requirement for a product because it affects the shelf life, color, WHC, and cooking yield of meat and meat products. No significant variations of pH were seen in the treatment groups compared to the control. Similar results were also reported by Ikhlas et al. [18]. ZnSO_4_ is a highly soluble ionic compound, to the point that it is a hydrate. Since it is so soluble, it will not chemically react with water to produce a product. The T4 group had a significantly higher WHC (*p* < 0.05) compared to the control. The WHC, also known as hydration capacity, water-binding capacity, or water absorption capacity, refers to the ability of proteins to retain water or the capacity per gram of protein to absorb water. The higher percentages of protein in T4 broiler meatballs might result in a higher WHC in the product. With the increase of Zn amount, the cook yield was gradually increased. The higher WHC of T4 meatballs resulted in a higher cook yield in this group. Furthermore, due to the good adhesive strength criteria of tapioca flour [19], the weight of all meatballs from their raw states may be increased.

### Sensory and texture analysis

**Figure 1.**
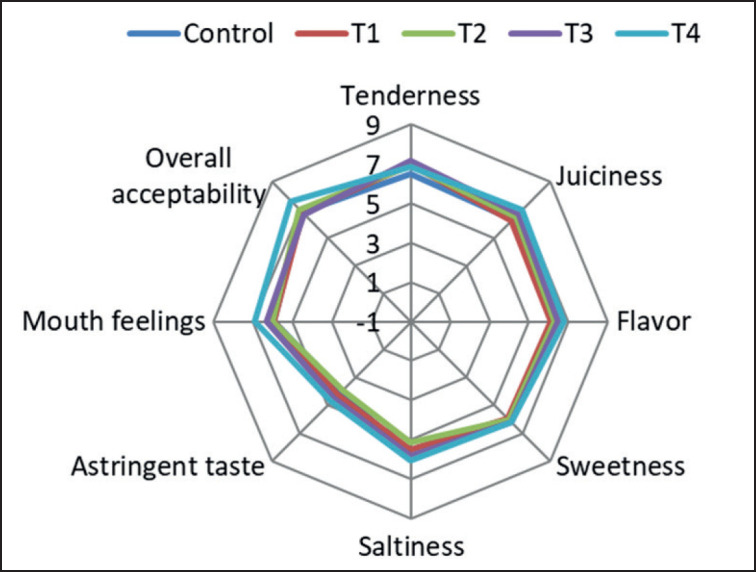
Radar chart representation of sensory test of control and Zn-fortified meatballs. T0–0 (control), T1–10, T2–20, T3–30, and T4–40 mg/kg. Eight distinct qualities are taken into account, including tenderness, juiciness, flavor, sweetness, saltiness, astringent taste, mouthfeel, and overall acceptability. The evaluation of each character was conducted using a nine-point scale ranging from 1 to 9, with 1 representing a favorable trait and 9 representing an unpleasant trait. The panelists did not perceive any significant differences among the various treatments.

According to Salgueiro et al. [20], ZnSO₄ can modify the sensory characteristics of several food items. That is why, in this study, the sensory qualities of Zn-fortified meatballs were assessed through a consumer panel. The results of the sensory analysis are presented in Figure 1. For identifying the instrumental value of product textural features, only the hardness value (Table 1) was tested. The hardness of the meatballs did not significantly change when different amounts of ZnSO_4_ were added. Even though the hardness values were not significantly different from the control, the increased hardness of all Zn-treated meatballs had the same effect on cheese, as Kahraman and Utsonol [16] reported. Probably, significantly higher protein in T4 meatball resulted in a higher hardness value in comparison to the control, though the panelists could not identify any significant differences. The panelist’s evaluation, as presented in Figure 1, further validated the instrumental findings regarding hardness presented in Table 1. Specifically, the juiciness and tenderness values assessed by the panelist did not exhibit any significant differences (*p* > 0.05). The relationship between juiciness and tenderness is typically attributed to the WHC of the product. Although the T4 group meatballs exhibited a significantly higher cook yield (*p* < 0.01) and WHC (*p* < 0.05), the panelists did not perceive any significant differences among the various treatments. However, the panelists assigned the highest overall level of approval to T4 meatballs. The levels of juiciness, flavor, sweetness, saltiness, and mouthfeel exhibited minimal variation between the Zn-treated samples and the untreated control samples, as depicted in Figure 1. The current sensory findings align with the findings of Abd-Rabou et al. [21], who similarly observed that the addition did not have a significant impact on the sensory characteristics of cheeses. The meatballs treated with varying concentrations exhibited comparable characteristics and were considered satisfactory by the panelists.

**Table 1.** Proximate composition and quality parameters of broiler meatball treated with different levels of ZnSO_4_.

Treatment (mg ZnSO_4_/kg meatball mix)							
Parameters	T0 (0 mg/kg)	T1 (10 mg/kg)	T2 (20 mg/kg)	T3 (30 mg/kg)	T4 (40 mg/kg)	SEM	*p-*value
Proximate composition							
Moisture (%)	72.68	72.00	71.28	71.97	74.53	0.929	0.881
Crude protein (%)	13.21^c^	13.09^bc^	12.31^bc^	14.39^b^	18.18^a^	0.603	0.000
Ether extract (%)	2.10	1.20	1.80	1.80	1.60	0.111	0.102
Quality parameters							
pH	5.81	5.89	6.08	5.88	5.86	0.039	0.23
Cook yield (%)	103.21^b^	103.20^b^	103.63^b^	107.60^a^	109.35^a^	0.600	0.000
WHC (%)	48.16^ab^	43.50^b^	49.92^ab^	48.00^ab^	52.58^a^	1.127	0.029
Hardness (*N*)	7.48	8.79	8.22	9.30	8.62	0.350	0.588

*p* < 0.01 = Significant at 1% level, *p *< 0.05 = Significant at 5% level, *p *> 0.05 = Nonsignificant. Figures with the same letter do not differ significantly, whereas figures with dissimilar letter differ significantly (as per DMRT).

### Determination of Zn

Zn content in foods was found to be varied with different processing methods [22]. Therefore, in the current study, the amount of Zn in meatballs was analyzed to see how much Zn was retained after cooking. The Zn content of meatballs that have been fortified with varying amounts of ZnSO₄ is displayed in Table 2. A minute quantity of Zn was detected in the cooked control groups; because it was below the levels of detection limit, the machine could not identify it. There were no significant differences seen in the cooked samples, though the T4 meatball exhibited the highest Zn content, suggesting a potential considerable variation from the control group. The raw and cooked T4 meatballs contained 30.67 and 16.72 mg/kg, respectively. The Zn recovery rates in T4 meatballs were found to be 41.8%, though the T3 meatballs recovered higher (49.06%) and the T2 meatballs recovered lower (35%) amounts of Zn than the T4 group. It was observed that one meatball weighs about 12 gm on average and has about 0.20 mg of Zn in each meatball. Therefore, 5 meatballs can provide 1-mg Zn. However, the highest tolerable level of Zn for the human body is 40 mg/d [23], and that is why it is completely safe.

### Storage quality

Several nonsensory indexes, such as thiobarbituric acid reactive substances (TBA), total volatile basic nitrogen, and TVC, are commonly used in assessing the freshness of food products [24,25]. The current study investigated the TBARS and TVC as indicators for assessing the freshness of meatballs under chilling and frozen storage conditions.

### TBA analysis

**Figure 2.**
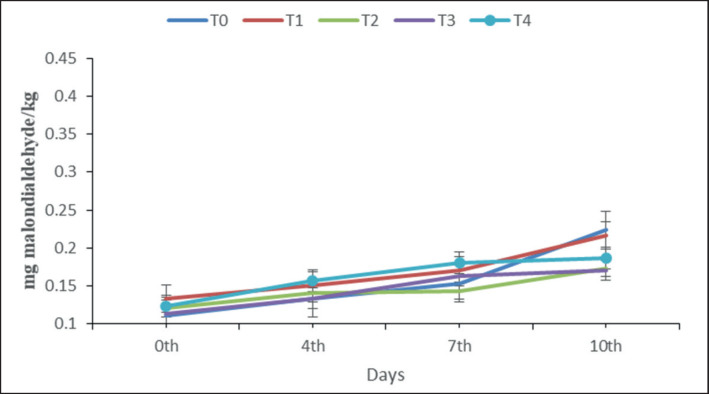
Effect of varying levels of Zn fortification on lipid oxidation (mg MDA/kg) of meatball during storage at 4°C–10°C for 10 days. T0–0 (control), T1–10, T2–20, T3–30, and T4–40 mg/kg. No significant changes were observed in the whole storage period.

The TBA assay is a widely employed method for quantifying lipid oxidation in food products. Lipid oxidation is an important quality parameter in the context of meat and meat products, as it has the potential to induce rancidity and serve as an indicator of product freshness [26]. According to the USDA Food Safety and Inspection Service, cooked meat is generally safe to consume when refrigerated for 3–4 days. Beyond this period, the meat may begin to lose its freshness. In this study, we extended the storage time to 10 days to assess the quality of fortified meatballs beyond the standard refrigeration period, providing insights into how they hold up under slightly prolonged storage conditions. In the present study, the MDA of all samples (Fig. 2) increased with the increase in the storage period. Although there were variations in the values observed on the fourth and seventh days, there were no visible changes in the meatballs among the different treatment groups at the end of the preservation period. During the duration of storage, the levels of MDA in all the samples remained within an acceptable range of 0.11 ± 0.02 to 0.22 ± 0.02. Kahraman and Ustonol [16] have documented comparable findings for the Zn-fortified cheddar cheese. According to the findings of Zago and Oteiza [27], it was observed that Zn has the ability to protect lipid oxidation in biological systems, hence enhancing the longevity of products. Consequently, the findings of the current investigation demonstrated low MDA levels as a result of the suppression of lipid oxidation within the food system. The control group did not exhibit any significant differences from other treatments, probably because of hygienic measurements followed throughout the processing, storage, and analysis. Various investigations have determined the recognized threshold for rancidity in meat and meat products is 2–2.5-mg MDA/kg [28], and the findings of our study indicated that all the samples examined were found to be below the threshold level. Thus, the present result demonstrates that meatballs fortified with Zn may be effectively stored within a temperature range of 4°C–10°C for a duration of 10 days.

**Table 2.** Amount of Zn and Zn recovery rate (% ) by broiler meatball treated with different levels of ZnSO_4_.

Treatment (mg ZnSO_4_/kg meatball mix)							
Parameters	T0 (0 mg/kg)	T1 (10 mg/kg)	T2 (20 mg/kg)	T3 (30 mg/kg)	T4 (40 mg/kg)	SEM	*p-*value
Amount of Zn (mg/kg) in raw meatball	3.83e	6.74d	11.60c	22.67b	30.67a	2.857	0.001
Amount of Zn (mg/kg) in cooked meatball	ND	ND	7.00	14.62	16.72	1.491	-
Zn recovery (%) by cooked meatball	ND	ND	35	49.06	41.8		

*p* < 0.01 = Significant at 1% level, *p* < 0.05 = Significant at 5% level, *p* > 0.05 = Nonsignificant. Figures with the same letter do not differ significantly whereas figures with dissimilar letter (a,b,c,d,e) differ significantly (as per DMRT).

### TVC analysis

**Figure 3.**
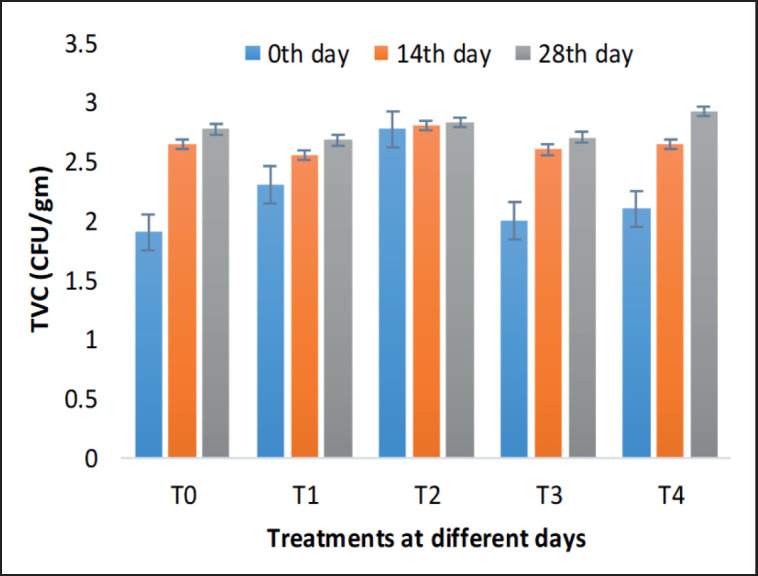
Effect of varying levels of Zn fortification on TVC of meatball during storage at −20°C for 28 days. T0–0 (control), T1–10, T2–20, T3–30, and T4–40 mg/kg. No significant changes were observed in the whole storage period.

Figure 3 displays the TVC observed in both the control and Zn-treated meatball samples. There was an absence of a significant amount of bacterial presence in the samples for the entire duration of preservation. In our study, the microbial load was always less than log 5 CFU/gm, but a bit started to increase from day 0 to 28 days during storage at −20°C. Generally, at temperatures ranging from −18°C to −20°C, all microbial activity is suspended if the necessary storage conditions are maintained. Additionally, Zn can interfere with microbial enzyme activity and further reduce microbial proliferation in food products [29,30]. It is essential to note that bacteria are not eliminated and will restart multiplying at their usual rate once the frozen food is thawed and left at room temperature. However, according to the findings of Kahraman and Ustunol [16,19], the addition of ZnSO_4_ at a concentration of 16 mg/kg to cheese milk did not exhibit any inhibitory impact on the starting culture.

Food-grade ZnSO_4_ monohydrate is commonly utilized for Zn fortification due to its cost-effectiveness. In this study, the elemental Zn content was calculated for each treatment group. Specifically, for the T4 treatment, 110 mg/kg of ZnSO_4_ was incorporated into the meatball formulation. Based on the current market price of food-grade ZnSO_4_, which is approximately $6 per kilogram (equivalent to 720 BDT/kg at the current exchange rate), the cost of 110 mg of ZnSO_4_ is approximately 0.08 BDT. This minimal cost is unlikely to have a significant impact on the per kg overall production expenses. Against this insignificant cost due to Zn fortification, it is expected to add substantial value to the product for the health benefit offered by Zn.

## Conclusion

By definition, food fortification is the practice of incorporating essential micronutrients, i.e., vitamins and minerals (including trace elements), in food irrespective of whether the nutrients were originally present in the food before processing or not, so as to improve the nutritional quality of food and to offer a public health benefit with minimal risk. A fortifier must not alter the food vehicle’s composition, texture, quality, and taste. In this study, broiler meatballs showed some positive changes in crude protein and WHC. However, the sensory properties of meatballs showed no significant differences between ZnSO_4_-treated and untreated samples. These results conclude that applying ZnSO_4_ can be a suitable approach for fortifying chicken meatballs and could be an excellent food source for improving Zn levels in groups at risk of Zn deficiency. This is the first-ever study on fortifying chicken meat products using ZnSO₄ in Bangladesh and elsewhere in the region. A continuation of this study, using animal models and human trials (e.g., Zn in blood plasma), is needed to understand better the bioavailability and absorption of Zn used in fortified meat products.

## List of abbreviations

DMRT, Duncan’s multiple range test; MDA, Malondialdehyde; SPSS, Statistical package for the social science; TBA, Thiobarbituric acid; TVC, Total viable count; WHC, Water-holding capacity; Zn, Zinc; ZnO, Zinc oxide; ZnSO_4_, Zinc sulfate.
